# Intratendinous Ganglion Cyst of the Hand: A Case Report and Review of the Literature

**DOI:** 10.1155/2020/8847563

**Published:** 2020-10-14

**Authors:** Byron Chalidis, Dimitrios Kitridis, Christos Dimitriou, Panagiotis Givissis

**Affiliations:** Aristotle University of Thessaloniki, 1st Orthopaedic Department, George Papanikolaou Hospital, Thessaloniki, Greece

## Abstract

Intratendinous ganglion of the hand is an extremely rare benign tumor, and only few cases have been reported so far in the literature. We present a case with an intratendinous ganglion of the extensor digitorum communis that treated with en bloc resection and subsequent tendon repair. According to the review of the literature and published data, the ganglion is predominantly located at hand extensor tendons (82%), and it is more frequent among females (75%) and shows a high incidence in 5^th^ and 6^th^ decades of life (94.5%). Surgical excision with or without side-to-side repair and/or tendon transfer leads to excellent outcome and low potential for recurrence.

## 1. Introduction

Intratendinous ganglion of the hand is a very rare entity, and only few cases have been reported so far in the literature [1–9]. The pathogenesis behind the intratendinous ganglion formation is still obscure, and various theories have been suggested [3]. It is believed that ganglion cysts may arise “internally” from mucoid degeneration of the tendon ground substance, forming cavities full of viscous fluid, or “externally” as the result of tendon invasion by tenosynovitis [1, 2, 7, 9]. Despite its benign character, the lesion can be locally aggressive and impair tendons and hand function. Symptom severity is related to the location of the ganglion, degree of tendon degeneration, and presence of associated synovitis [3, 8]. Although the tumor usually affects the extensor tendons, other uncommon sites of involvement have been described [3, 5, 6, 8]. Apart from presenting a case with intratendinous dorsal wrist ganglion with a long-term follow-up, we systematically reviewed the available literature and tried to evaluate the patients and tumor characteristics as well as the clinical outcome of surgical treatment.

## 2. Case Report

**Figure 1:**
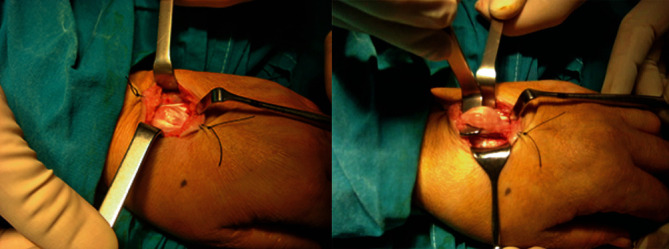
Intraoperative picture showing intratendinous lesion of the EDC tendon.

**Figure 2:**
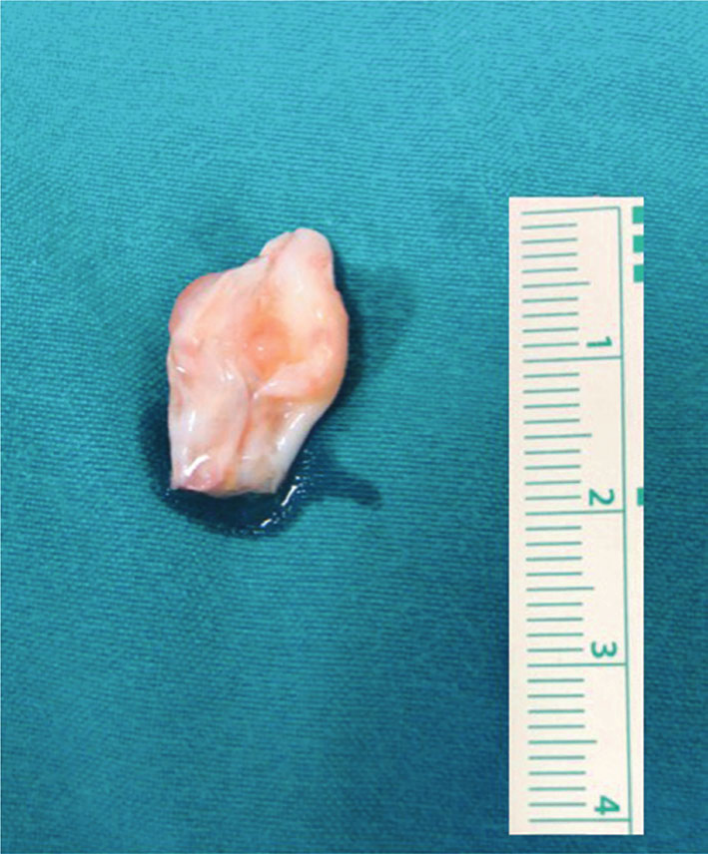
Intraoperative specimen of the excised tumor including a portion of the EDC tendon substance.

We present the case of a 47-year-old, right-hand dominant woman who was referred to our department with a 6-month history of a palpable soft-tissue mass at the dorsal aspect of the left hand that caused progressive pain in finger movements. A magnetic resonance imaging scanning illustrated a well-defined hourglass-shaped ganglion cyst in direct contact to the middle finger extensor digitorum communis (EDC) tendon. Under regional anesthesia, a longitudinal dorsal hand approach was utilized, and a cyst mass of approximately 10 mm × 20 mm with jelly-like contents adhered to EDC was identified (Figure 1, Video 1). The ganglion along with the surrounding degenerative tendon tissue was excised en bloc (Figure 2). As approximately half of the middle finger extensor tendon retained after tumor resection, simple repair of the remaining tendon was applied using three 4-0 absorbable sutures. Histologic examination confirmed the cyst nature of the lesion. Postoperatively, the wrist was immobilized in a short-arm splint for 4 weeks with the wrist in 20 degrees of extension, the metacarpophalangeal joints in 30 to 40 degrees of flexion, and the interphalangeal joints in full extension. After that period, progressive wrist and hand mobilization were commenced. The patient returned to her regular duties as a housewife after 3 months. At the latest follow-up, 10 years postoperatively, she was symptom-free, and no evidence of tumor recurrence was apparent. No flexion deficit or extension lag of the involved middle finger was recognized, and she could perform all the activities of daily living without any restriction.

## 3. Discussion

**Figure 3:**
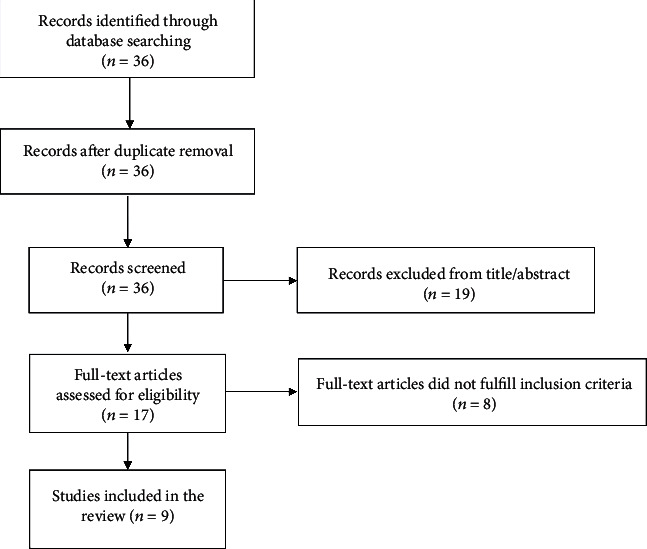
Flowchart diagram for inclusion and exclusion of papers.

According to the Preferred Reporting Items for Systematic Reviews and Meta-Analyses (PRISMA), a systematic search of the Medline database up to May 2020, using the keywords “intratendinous AND ganglion”, was performed by two independent investigators, B.C. and D. K [10]. Clinical studies relevant to intratendinous ganglions of the hand were eligible for the review. The initial search revealed 36 records, and nine articles were included in the review (Figure 3). One article was level IV evidence [8] and eight articles level V [1–7, 9]. The same independent investigators extracted data from the studies for the final descriptive data synthesis.

Ten studies, including our case, revealed 18 patients with 22 intratendinous hand ganglia (Table 1) [1–9]. Women are affected more frequent than men (75% Vs 25%). It is worth noting that 18 out of 19 patients had an age between 43 and 66 yrs (94.5%), and only one patient was 71 yrs old. Regarding the tumor location, the vast majority was appeared in extensor tendons (18 cases, 82%) [1, 2, 4–9]. Apart from EDC (14 cases) [1, 4, 5, 7, 8], the ganglion cysts were affected by the extensor pollicis longus (EPL) (2 cases) [5, 6] and the extensor pollicis brevis (EPB) (2 cases) [2, 9]. The remaining 4 ganglia (18%) were identified in abductor pollicis longus (AbPL) (1 case) [7], adductor pollicis longus (AdPL) (one case) [7], and flexor tendons [one case in flexor digitorum superficialis (FDS) and one in flexor digitorum profundus (FDP)] [3, 8]. Flexor tendon cysts may limit finger extension and lead to stiffness [3, 7]. Senda et al. [7] noted that as flexor tendon ganglia were located in the deep layer of the hand, they could be diagnosed quite late until they became large enough. Therefore, latent asymptomatic flexor ganglions probably might occur more frequently than reported. Recently, Botchu et al. [1] reported also a case with iatrogenic intratendinous ganglion cyst of the EDC tendon of the middle finger following intravenous cannulation. They postulated that the EDC of the middle finger was injured during attempted venepuncture, and awareness of this unusual pathology should be considered in the evaluation of a dorsal wrist lump.

In 8 out of the 10 presented articles, the follow-up was short (range, 2 mon to 2.5 yrs) and therefore, no definite conclusion can be drawn regarding the possibility of tumor recurrence [1–6, 8, 9]. Seidman and Margles [7] reported one recurrence at 5.5 years postoperatively (4.5%). In our case, no tumor reappearance was detected at 10 yrs postoperatively. Although it seems that complete excision of ganglion offers potentially curative treatment, long-term follow-up is essential for evaluating the potential for recurrence. In the vast majority of presented cases, tumor removal was associated with partial or incomplete tendon tear, and subsequent repair was utilized with 4-0 or 5-0 absorbable sutures. The use of absorbable sutures was proved adequate to stabilize the repair site and facilitate early mobilization of the affected tendon as no failures were reported. However, in some circumstances, en bloc resection of the tumor may leave only a small portion of intact tendon and cause significant weakness of the tendon structure. In this scenario, primary repair is not feasible, and tendon reinforcement by the side-to-side procedure or even tendon transfer should be performed to restore hand function [6, 8]. Satonaka et al. [6] transferred the extensor indicis proprius (EIP) tendon to the distal stump of EPL to restore the extension of the thumb after en bloc excision of EPL intratendinous ganglion. Nevertheless, in all published cases, neither postoperative tendon rupture nor further operations were undertaken.

## 4. Conclusion

According to the current presented case and literature review, complete intratendinous ganglion excision leads to excellent result with low potential for recurrence. The diagnosis should be suspected mainly in female patients in the 5th or 6th decade of life suffering from a painful palpable mass within extensor tendons of the wrist but uncommon presentation in the dorsal thumb or flexor tendons could not be ruled out.

## Figures and Tables

**Table 1:** Studies and patients characteristics.

Study	Year	Patients/cases	Tumor location	Follow-up	Gender(M/F)	Age (Yrs)	Treatment
Seidman and Margles [7]	1993	7/10	EDC (9 cases) AdPL (1 case)	Mean 6 yrs (2 Mon to 12 yrs)	2M/5F	Mean 55 (range, 42-66)	Excision (9 cases) Excision-side to side repair (1 case)
Ikeda et al. [4]	2001	1	EDC	2.5 yrs	M	53	Excision-repair
Chew et al. [2]	2010	1/2	EPB and AbPL	NA	F	43	Excision-repair
Chia et al. [3]	2015	1	FDP (little finger)	1 year	F	73	Excision-repair
Satonaka et al. [6]	2015	1	EPL	3 months	F	45	Excision-EIP transfer to EPL
Lee et al. [5]	2015	2	EPL and EDC	2 months-2 years	2F	51 and 51	Excision-repair
Young and Freiberg [9]	2015	1	EPB	NA	M	51	Excision-repair
Senda et al. [8]	2017	2	FDS (ring finger) and EDC	2 years	N/A	56 and 58	Excision-side to side repair
Botchu et al. [1]	2018	1	EDC	NA	F	50	Excision-repair
Chalidis	2020	1	EDC	10 years	F	47	Excision-repair

## Supplementary Materials

Supplementary Materials Video 1: intraoperative video showing intratendinous ganglion of the middle finger extensor digitorum communis (EDC) tendon.
